# Evolutionary and genomic insights into the long-term colonization of *Shigella flexneri* in animals

**DOI:** 10.1080/22221751.2022.2109514

**Published:** 2022-08-31

**Authors:** Junrong Liang, Zhen Zhu, Ruiting Lan, Jing Meng, Bram Vrancken, Shan Lu, Dong Jin, Jing Yang, Jianping Wang, Tian Qin, Ji Pu, Li Zhang, Kui Dong, Mingchao Xu, Huaiyu Tian, Taijiao Jiang, Jianguo Xu

**Affiliations:** a State Key laboratory of Infectious Disease Prevention and Control, National Institute for Communicable Disease Control and Prevention, Chinese Center for Disease Control and Prevention, Beijing, People’s Republic of China; b College of Life Science and Food Engineering, Hebei University of Engineering, Handan, People’s Republic of China; c School of Biotechnology and Biomolecular Sciences, University of New South Wales, Sydney, Australia; d Center for Systems Medicine, Institute of Basic Medical Sciences, Chinese Academy of Medical Sciences & Peking Union Medical College, Beijing, China; e Suzhou Institute of Systems Medicine, Suzhou, People’s Republic of China; f Department of Microbiology and Immunology, Rega Institute, Laboratory of Evolutionary and Computational Virology, KU Leuven, Leuven, Belgium; g Scientific research department, Shanghai Public Health Clinical Center, Fudan University, Shanghai, People’s Republic of China; h Research Units of Discovery of Unknown Bacteria and Function, Chinese Academy of Medical Sciences, Beijing, People’s Republic of China; i Shanxi Eye Hospital, Taiyuan, People’s Republic of China; j State Key Laboratory of Remote Sensing Science, Center for Global Change and Public Health, College of Global Change and Earth System Science, Beijing Normal University, Beijing, People’s Republic of China; k Guangzhou Laboratory, Guangzhou, People’s Republic of China; l Research Institute of Public Heath, Nankai University, Tianjin, People’s Republic of China

**Keywords:** *Shigella flexneri*, phylogenetic analysis, SNP typing, animal reservoir, evolution

## Abstract

The enteroinvasive bacterium *Shigella flexneri* is known as a highly host-adapted human pathogen. There had been no known other reservoirs reported until recently. Here 34 isolates obtained from animals (yaks, dairy cows and beef cattle) from 2016 to 2017 and 268 human *S. flexneri* isolates from China were sequenced to determine the relationships between animal and human isolates and infer the evolutionary history of animal-associated *S. flexneri*. The 18 animal isolates (15 yak and 3 beef cattle isolates) in PG1 were separated into 4 lineages, and the 16 animal isolates (1 yak, 5 beef cattle and 10 dairy cow isolates) in PG3 were clustered in 8 lineages. The most recent human isolates from China belonged to PG3 whereas Chinese isolates from the 1950s–1960s belonged to PG1. PG1 *S. flexneri* may has been transmitted to the yaks during PG1 circulation in the human population in China and has remained in the yak population since, while PG3 *S. flexneri* in animals were likely recent transmissions from the human population. Increased stability of the large virulence plasmid and acquisition of abundant antimicrobial resistance determinants may have enabled PG3 to expand globally and replaced PG1 in China. Our study confirms that animals may act as a reservoir for *S. flexneri*. Genomic analysis revealed the evolutionary history of multiple *S. flexneri* lineages in animals and humans in China. However, further studies are required to determine the public health threat of *S. flexneri* from animals.

## Introduction

*Shigella* is a member of the family *Enterobacteriaceae*, and is the primary agent of shigellosis, or bacterial dysentery, which mostly affects children under the age of five years [1,2]. This bacterium has evolved from *Escherichia coli* multiple times, through parallel acquisition of key virulence factors including the invasion plasmid pINV to become pathogenic [3]. *Shigella* currently stands as a genus with 4 species [4]. *Shigella flexneri* is predominant in low and lower middle-income countries, accounting for more than 50% of all cases of shigellosis in these countries, whereas *S. sonnei* mainly occurs in high-income countries [5–7]. Complex factors contribute to the successful spread of *Shigella*, including unreliable sanitation systems, unavailability of clean drinking water, the ability of the organism to invade and subvert host defences, and the acquisition of antimicrobial resistance (AMR) [2,5]. *Shigella* pathogenesis-associated genomic regions are chromosome pathogenicity islands (PAIs) and the large virulence plasmid (pINV) [8–10]. pINV carries a 37 kb “entry region” that encodes the Type III secretion system (T3SS) and the T3SS effectors which are essential for *Shigella* the bacteria to infect host cells [11,12]. Chromosome-encoded pathogenicity islands SHI-1 and SHI-2 also play an important role in virulence [9,13,14].

*S. flexneri* shows considerable diversity with more than 17 recognized serotypes [15], and serotype 2a predominates in endemic countries [16]. The most *S. flexneri* circulating in China belonged to sequence type (ST) 91 with serotypes 2a and Xv prevalent [17,18]. A global genomic study divided the *S. flexneri* population into 7 phylogenetic groups (PGs) [19]. These different PGs were found in all geographic regions with some geographic restrictions. Although *Shigella* is known as a highly host-adapted human pathogens [5], a recent study reported the isolation of *S. flexneri* from animals [20,21]. In this study, we isolated and performed genome sequencing of 34 *S. flexneri* isolates obtained from animals (yaks, dairy cows and beef cattle) from 2016 to 2017 and 268 human *S. flexneri* isolates from China. We further discussed the relationships between the animal- and human -associated *S. flexneri* and inferred their evolutionary history. Our work contributes to the development of appropriate measures for the surveillance and control of *S. flexneri* infections.

## Materials and methods

### Sampling from animals and isolation of *S. flexneri*

Samples from dairy cows and beef cattle were collected from the Gansu, and Shanxi provinces during 2016–2017. Domestic yaks were sampled from Qinghai and Gansu provinces of the Qinghai–Tibet Plateau. Rectal swab were the preferred sampling choice, followed by the faecal samples. An improved method for traditional isolation and culture was developed. Anaerobic culture enrichment was performed using *Shigella* broth (SB) with 0.5 μg/ mL novobiocin at 37°C for 8–10 h (0.2 ml of each sample was inoculated into 10 mL of SB). All the enriched samples were screened for *Shigella* using a PCR assay targeting the *ipaH* gene [22]. The *ipaH* PCR positive samples were inoculated onto 2 different types of selective agar medium media (Hektoen enteric agar (HEA) medium and xylose lysine deoxycholate (XLD) agar medium, Oxoid, Basingstoke, UK) to detect *Shigella* spp. [23]. Typical colonies were selected and recultured on brain heart infusion (BHI) agar plates (Oxoid, UK) at 37 °C for 24 h.

### Shigella flexneri human-associated isolates

In total of 268 human isolates from China were selected including 7 human isolates from the 1950s to 1960s and 261 human isolates from 1997 to 2017, 59 of which were sequenced in a previous study [17]. These isolates with different serotypes were collected from 17 provinces of China. As it was the major cause of shigellosis in China since 2000, more isolates of serotype Xv were selected. We also included 346 genome sequences of human isolates from public databases from Africa, Asia, Latin America, Europe, and North America (Publicly available at the NCBI BioProject (PRJEB2846, PRJEB2460 and PRJEB2542)) [19]. The isolates used in this study are listed in supplementary Table S1.

### Whole genome sequencing and detection of single nucleotide polymorphisms (SNPs)

DNA was extracted using the Wizard® Genomic DNA Purification Kit (Promega, USA) according to the manufacturer’s instructions. Library preparation was performed using the Nextera XT Library Prep Kit (Illumina, USA) according to the manufacturer’s instructions. The libraries were sequenced on an Illumina/Solexa platform with a minimum 100-fold coverage at the Tianjin Biochip Corporation (Tianjin, China).

After quality control and read trimming using FastQC [24], the high-quality reads were assembled using Spades 3.13.0 [25]. Sequencing reads were mapped to the reference genome (*S. flexneri* serotype xv strain 2002017: accession number: CP001383.1) using BWA v0.7.17 with the default settings. Snippy v4.4.5 was used to call SNPs between the reads and reference genome with the default parameters: snippy -cpus 16 -outdir mysnps -ref 2002017.gbk -R1 R1.fastq.gz -R2 R2.fastq.gz (https://github.com/tseemann/snippy) [26].

### Construction of phylogenetic relationships

To mitigate the effect of recombination on phylogenetic analyses, we identified the recombinant regions with high SNP densities using Gubbins v3.0.0 [27], and removed them for phylogenetic analysis of the 648 *S. flexneri* isolates (34 animal isolates, 268 Chinese isolates sequenced by us and 346 publicly available genomes including 4 from mainland China). To infer a maximum likelihood tree based on the non-recombinant genome region SNPs, we ran FastTree v2.1.11 with the generalized time-reversible model and a gamma distribution for modelling the site rate variation[28]. We estimated the phylogenetic patterns with 100 bootstrap replicates. To further determine the shared patterns of sequence variation, we used fastbaps v1.0.6, which is based on hierarchical Bayesian clustering, to partition the phylogeny into phylogenetic groups (PGs) [29].

### Temporal analysis

To assess the timing of divergence among *S. flexneri* isolates, we performed molecular clock analysis using BEAST2 v2.6.3 [30,31]. All the strains were used to capture the complete temporal and geographical range. BEAST2 was run independently across at least 2 chains of 100 million generations, each of which was sampled at every 1000 iterations to ensure agreement. Maximum likelihood trees constructed using PhyML v.3.0 [32] or Iqtree v.1.6.9 [33] were used for the initial evaluations of the presence of temporal signals in the dataset by regressing the root-to-tip distances versus sampling years in TempEst [34]. All Bayesian phylogenetic inferences were performed using BEAST 1.10 [35] and BEAGLE v.3 [36]. We used a GTR + Gamma substitution model [37], and the skygrid model [38] was specified as a flexible-tree prior. In the exploratory analyses, branch lengths were rescaled into time units using a strict or relaxed [39] clock model. A host-specific local clock model [40,41] was used to test for a host effect on the evolutionary rate. As it was unclear to which host category (human or animal) the branches basal to animal clades should be assigned, we integrated over both alternatives using a model averaging procedure to evaluate support for the hypotheses [42].

Convergence and mixing properties were evaluated in Tracer v1.7 [43], which was also used to determine the appropriate number of samples to be discarded as burn-in. Maximum clade credibility (MCC) summary trees were obtained using TreeAnnotator distributed in BEAST v1.10. The trees were visualized either using FigTree v.1.4.4 (https://github.com/rambaut/figtree) or iTol [44].

### Virulence factors and antimicrobial resistance determinants

The presence of virulence genes located in pINVs and PAIs and the AMR genes were identified using BLAST against the reference loci described in the VFDB [45], PAIDB v2.0 [46] and CARD databases [47] (CARD; https://card.mcmaster.ca). The virulence plasmid pCP301 of *S. flexneri* 2a str. 301 was used as the reference (accession number: AF386526.1).

### Analysis of host-associated accessory genes

The assembled contigs of each isolate were annotated using the Prokaryotic Genome Annotation System (Prokka) pipeline v1.14.5 [48]. The resulting annotations of all the isolates in GFF3 format were fed into the Roary v3.13.0 pangenome pipeline to identify the core and accessory genes [49]. We chose a percentage identity of 95% to distinguish the core genes from the accessory genes. Scoary v1.6.16 was used to determine the accessory genes that were associated with humans and animals [50]. We further confirmed the presence of putative animal-associated accessory genes in both animal and human isolates using BLASTN v2.11.0 [51].

## Results

### The serotype distribution of *S. flexneri* in China

**Figure 1.**
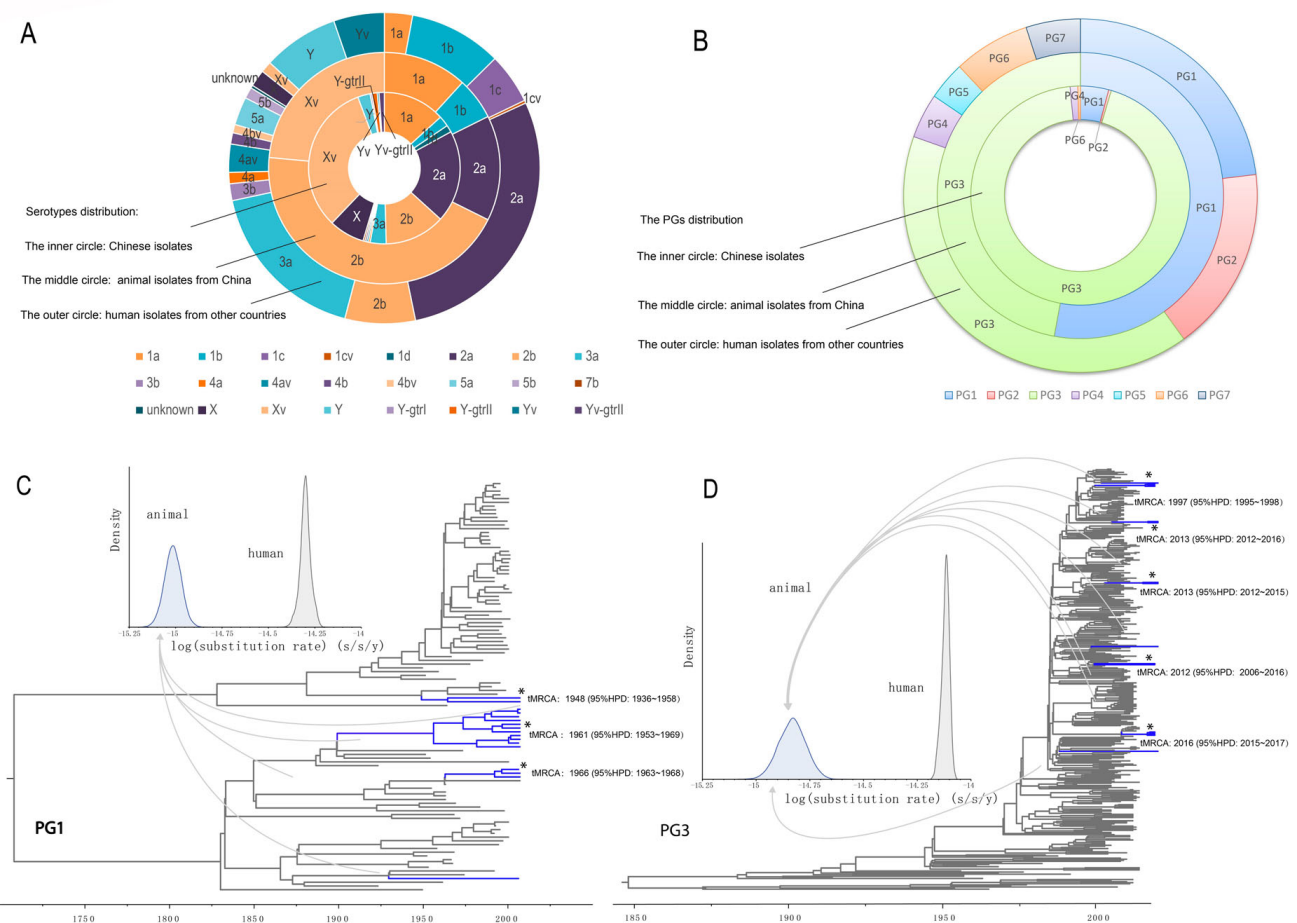
Characteristics and phylogenetic groups distribution, and maximum clade credibility (MCC) trees of PG1 and PG3 clades. A. Serotype distribution of the isolates used in this study. The inner, middle, and outer circles depict the proportion of human isolates from China, animal isolates, and human isolates from other countries by serotype. Serotypes were based on genome sequence predictions. Y-gtrI, Y-gtrIII and Yv-gtrII denotes the *gtrI, gtrIII* and *gtrII* genes detected in these isolates. B. Phylogenetic group (PG) distribution of the isolates used in this study. The inner, middle and outer circles depict the proportion of human isolates from China, animal isolates, and human isolates from other countries by the PGs. C: Maximum clade credibility (MCC) tree of PG1 isolates. The branches of animal isolates lineages are coloured in blue. For details of the phylogenetic relationships among the isolates, see supplementary figure S1. D: Maximum clade credibility tree of PG3 isolates. The branches of animal isolate lineages are coloured in blue. For details of the phylogenetic relationships of the isolates, see supplementary Figure S2.

The 34 *S. flexneri* isolates were obtained from 2,102 samples screened, with 16 from yaks, 10 from dairy cattle, and 8 from beef cattle. The detection rate was 1.22% (16/1311) in yaks, 2.11% (10/474) in dairy cows, and 2.52% (8/317) in beef cattle. Most isolates (61.76%, 21/34) were collected from calves with diarrhoea, with 6 from yak calves and 15 from other calves. Five different serotypes (2b, 2a, 1b, 1a and Xv) were identified among these animal-associated isolates, with serotype 2b being the most common, followed by serotype Xv (Figure 1(A)). The 268 human isolates from China belonged to 17 serotypes, with serotype Xv being the most predominant (32.09%) (Figure 1(A)).

### Phylogenetic structure of *S. flexneri*

Phylogenomic analysis revealed that the 648 *S. flexneri* isolates were divided into the previously defined 7 PGs reported by Connor et al. [19] (Figure 1(B), 2A). The 272 human isolates from China (268 sequenced by us and 4 from public database) were clustered into 5 PGs. Most isolates fell into PG3 (91.54%, 249/272), as the majority of the isolates were from 2001 to 2012. Only 8.46% of the isolates were clustered into other PGs (6.62% in PG1, 1.10% in PG4, 0.37% in PG2, and 0.37% in PG6). No Chinese isolates were found in PG5 and PG7 (Figure 1(B)).

Isolates from animal hosts were found in 2 PGs, with 52.94% (18/34) in PG1 and 47.06% (16/34) in PG3 (Figure 1(B)). The 18 animal isolates (15 yak and 3 beef cattle isolates) in PG1 were separated into 4 lineages (Figure 1(C)) with each containing one (serotype 1a), two (both serotype 1b), four (all serotype 2b), and 11 (all serotype 2b) isolates respectively and were not identical to any human isolates (Figure S1). The only serotype 1a animal isolate was closest to a 1978 France serotype 1a isolate; the 2 serotype 1b isolates were grouped together with three Chinese serotype 1b isolated isolated between 1997 and 2003 and one Chinese serotype 3a isolate from 2001; the four serotype 2b isolates grouped together with a 1965 serotype 2b human isolate from China and the 11 serotype 2b isolates were grouped with two 1950s serotype 2b human isolates from China (Figure S1). The 16 animal isolates (1 yak, 5 beef cattle and 10 dairy cow isolates) in PG3 were clustered in 8 lineages (Figure 1(D)). Five lineages contained two to four isolates wheras three lineages contained a single isolate. The animal lineages were grouped with different Chinese isolates sequenced in this study. Most PG3 animal isolates were obtained from Gansu province. Interestingly they were not grouped with human isolates from the Gansu province. There are 4 animal isolates from 2 different province grouped together (one from Gansu and three from Shanxi), and were closer to each other than to human isolates from Gansu (Figure S2).

### Temporal signals and evolutionary rates in the different host populations

**Figure 2.**
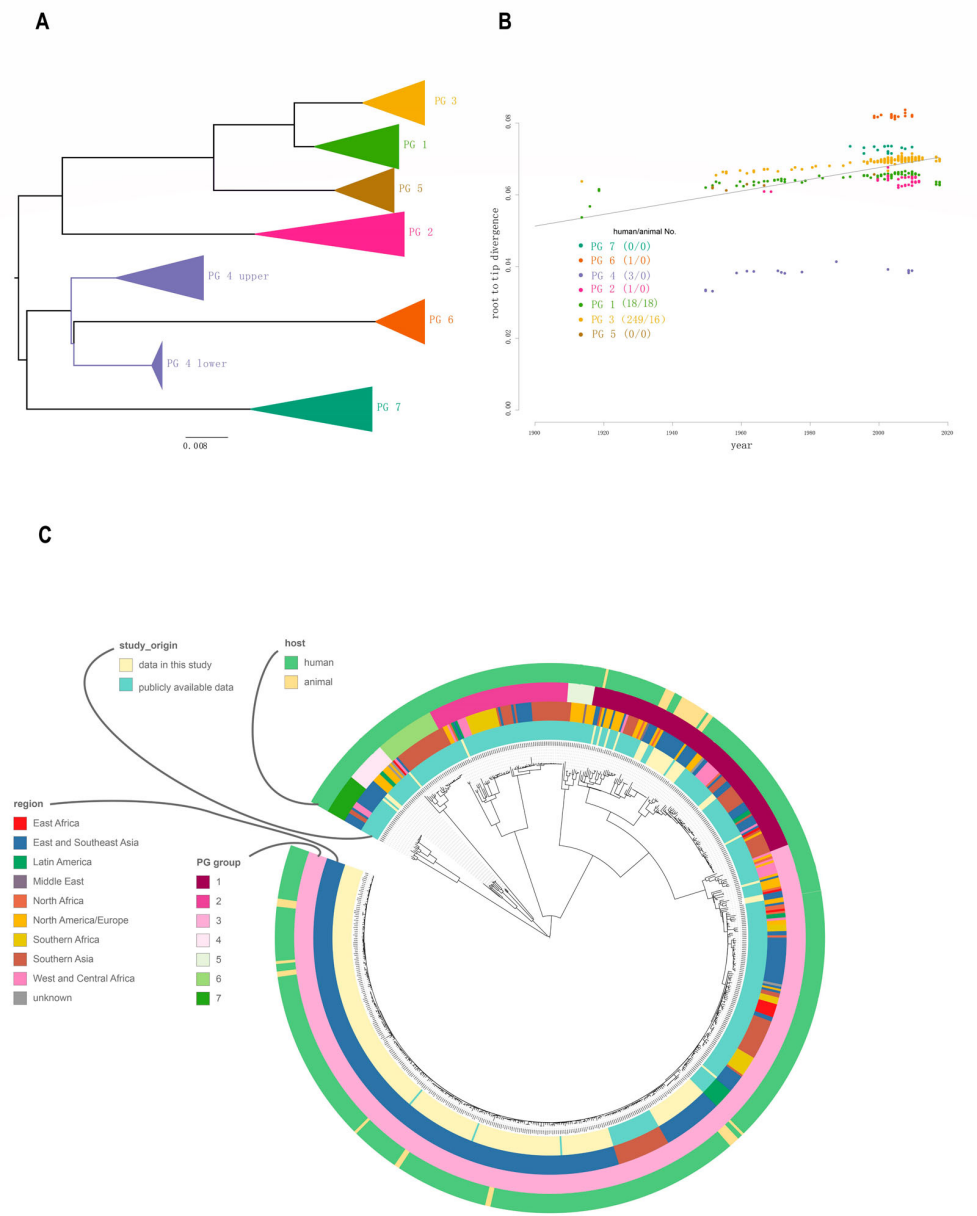
Population structure of the 648 *S. flexneri* isolates. A: PGs of the 648 *S. flexneri* isolates. The PGs were identified from the maximum likelihood tree of the 648 isolates and nodes within the PGs are collapsed. B: Exploration of the temporal signal in the data by regressing root-to-tip genetic distances against sampling times with residuals coloured by PG. Correspondence between the colours of the residuals and PGs is as indicated in the legend. The numbers between brackets indicate the strains obtained for this study in China from human and animal hosts, respectively for which information regarding sampling time was available. C: Maximum-likelihood tree of *S. flexneri* inferred from 61,581 single nucleotide polymorphisms (SNPs). All SNPs were recorded by their position in reference to the 2002017 genome. Potential genome-wide SNPs outside the recombinant regions were used. Coloured rings from the inside to outside indicate the study origin, geographical regions, and PG groups and hosts.

**Figure 3.**
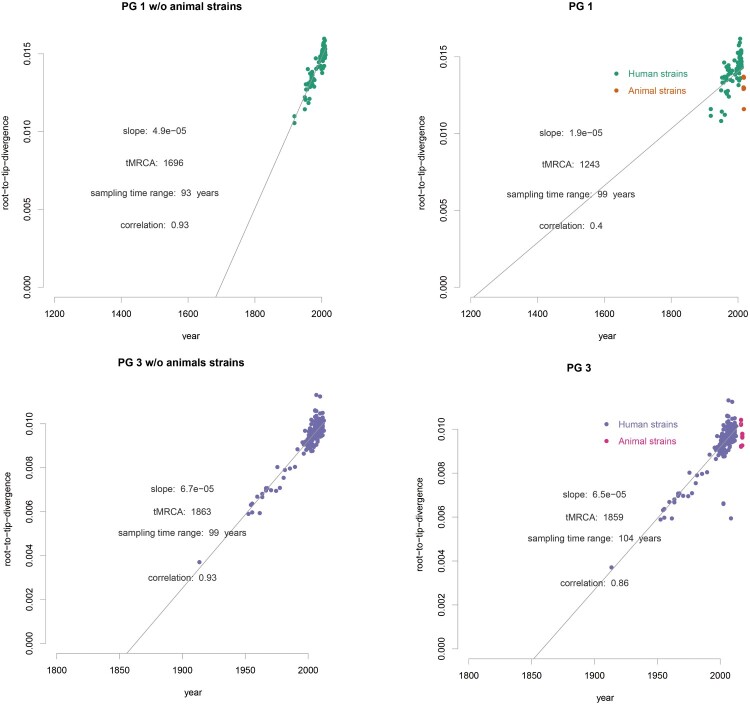
Clade-wise exploration of the temporal signals of PG1 and PG3 Root to tip divergence was computed using TempEST, with and without the animal isolates for PG1 and PG3 considered separately. The animal and human isolates are coloured as shown.

Using BEAST [30], we performed molecular clock analysis to estimate the time to the most recent common ancestor (tMRCA) of isolates from different animal lineages within PG1 and PG3 as all animal isolates and the most humane isolates in China fell within these 2 two clades (Figure 2(B,C)). Using the date of isolate collection to calibrate the respective phylogenies, the temporal signal varied among clades (Figure 2(B)). We computed the root to tip divergence using TempEST [34] and obtained a strong correlation between the time of sampling and the root-to-tip distances (Figure 2(B)), indicating a strong temporal signal for molecular clock analysis. The correlations were highly significant following 10,000 date randomizations (PG1: R2 = 0.4; PG3: R2 = 0.86). The residuals does not vary much without animal strains (without animal R2 = 0.93, with animal and human R2 = 0.86) in PG3 than in PG1 (without R2 = 0.93, with R2 = 0.4) (Figure 3). However, there was no correlation of root to tip divergence and sampling time for animal only isolates (data not shown). The evolutionary rates for PG1 and PG3 total isolates (human and animal isolates) were 6.0×10^−7^, (95% HPD: 5.8×10^−7^−7.3×10^−7^) and 6.1×10^−7^ (95% HPD: 5.9×10^−7^−6.3×10^−7^) substitutions per nucleotide site per year, respectively. For PG1 and PG3 human only isolates were 5.7×10^−7^ (95% HPD: 5.3×10^−7^–6.2×10^−7^) and 7.3×10^−7^ (95% HPD: 7.1×10^−7^–7.6×10^−7^). There was marginal difference in the rates with and without animal isolates (Figure 3). We also calculated evolutionary rates for PG1 and PG3 animal isolates only which were PG1: 9.7×10^−9^ (95% HPD: 5.7×10^−9^–1.4×10^−8^) and 3.4×10^−8^ (95% HPD: 1.5×10^−8^–5.5×10^−8^). The rates of animal only isolates were significantly slower than those of the human only isolates. Based on the evolutionary rate for the total human and animal isolates, the age (year) of the most recent common ancestor (tMRCA) for each of the 4 animal lineages within PG1 ranged from 1948 to 1966 (Figure 1(C), S1) and that for the 8 animal lineages within PG3 ranged from 1997 to 2016 (Figure 1(D), S2).

### Virulence factors and antimicrobial resistance determinants in different PGs and animal-associated isolates

**Figure 4.**
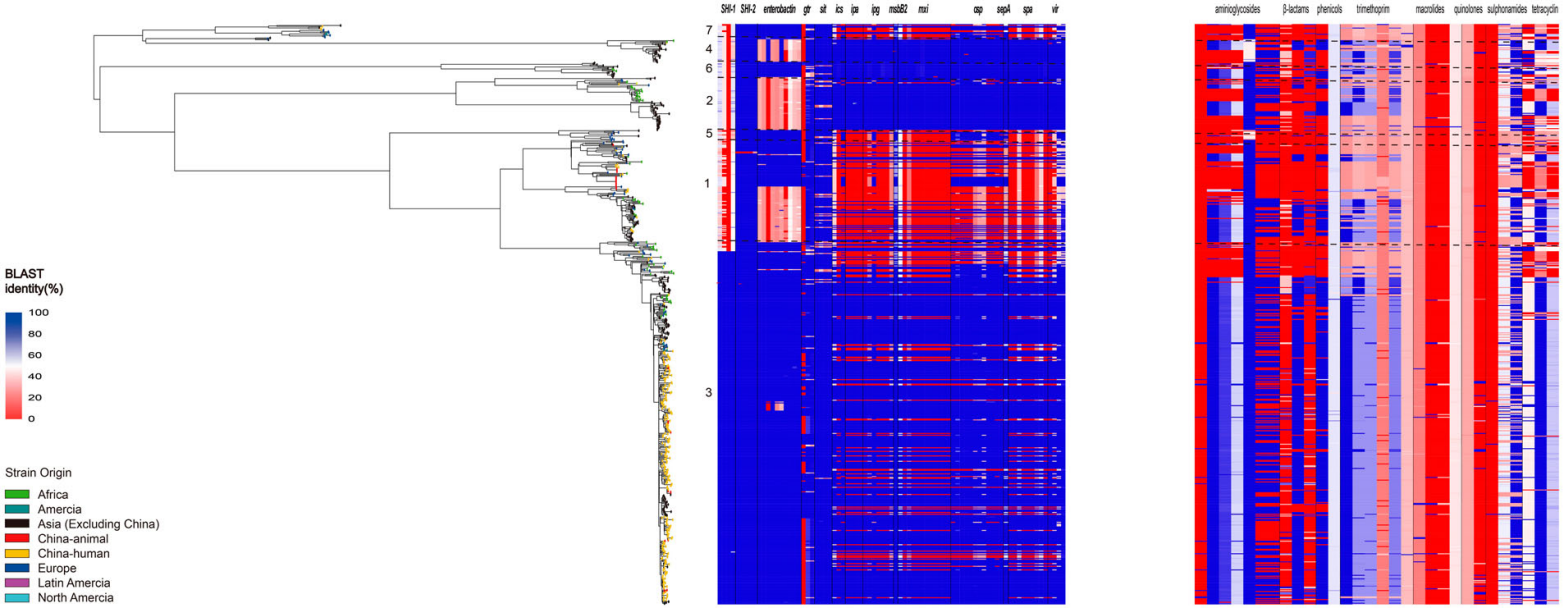
Comparison of the distribution of virulence factors and antimicrobial resistance determinants of *S. flexneri* isolates from animals and Chinese human isolates within and between PG1 and PG3. The compositions of virulence factors and AMR determinants in each isolate are represented as the percentage identity of the best BLAST hit. The virulence factors/genes (in order) are SHI-1 (pic, set1A, set1B, and sigA), SHI-2 (iucA, iucB, iucC, iucD, and iutA), enterobactin genes (entA, entB, entD, entE,entF, fepA, fepB, fepC, fepD, and fepG), sit (sitA, sitB, sitC, and sitD), ics (icsA/virG, icsB, and icsP), ipa (ipaA, ipaB, ipaC, and ipaD), ipg (ipgA, ipgB1, ipgB2, ipgC, ipgD, ipgE, and ipgF), msbB2, mxi (mxiA, mxiC, mxiD, mxiE, mxiG, mxiH, mxiI, mxiJ, mxiK, mxiL, mxiM, and mxiN), osp (ospB, ospC1, ospC2, ospC3, ospC4, ospD1, ospD2, ospD3, ospE1, ospE2, ospF, and ospG), sepA, spa (spa13, spa15, spa24, spa29, spa32, spa33, spa40, spa47, and spa9), and vir (virA, virB, virF, and virK). The AMR genes are aac(3)-II, aadA1, aadA2, aadA5, strA, strB, and sat1 (aminoglycosides); bla_CTX-M-24_, bla_OXA-1_, and bla_TEM-1_ (β-lactams); catA1 and catB1 (phenicols); dfrA17, dfrA3b, dfrA1, dfrA5, dfrA14 and dfrA8 (trimethoprims); ermB, msrE, mphA,and mphE (macrolides); qacEΔ1 and qnrS1(quinolones); qepA, sul1, and sul2 (sulphonamides), and tetA(A), tetA(D), and tetA(B) (tetracyclines).

The virulence plasmid pINV which carries a 37 kb entry region encoding the Ipa-Mxi-Spa type III secretion system (T3SS) is essential for *Shigella* virulence. It enable the bacterium to invade intestinal epithelial cells, escape into the host cell to move in the cytosol of infected cells, undergo cell-to-cell spread, and result in pyroptosis in macrophages [11,12]. The presence of pINV in the 648 isolates across different PGs was markedly different (Figure 4). Within PG 1 and PG3, the distribution of virulence factors between animal isolates and human isolates was similar (Figure S3). pINV was absent in 90.91%, 82.61% and 64.71% of the PG5, PG1 and PG4 isolates respectively, while pINV was not detected in 0%, 0%, 1.69% and 18.06% of the PG7, PG6, PG2 and PG3 isolates respectively. All PG1 isolates from animal sources harboured the 37 kb T3SS “entry region” deleted from the pINV. For other virulence factors, SHI-1 was mainly carried by PG3 isolates, and there was no difference in the presence of SHI-1 between human and animal PG3 isolates. The enterobactin iron acquisition system genes were widely present in PG3, but absent in 64.95% of PG1 isolates. The *sit* gene was occasionally absent in some human isolates, but was present in all animal isolates (Figure 4).

We also screened for all AMR genes using BLAST against the CARD databases. PG3 isolates carried significantly more AMR genes on average than those in PG1 isolates (Figure 4, S3), with the inter-quartile range (IQR) of 53.53% for PG3 compared to an IQR of 25.50% for PG1. However, the carriage rate of resistance genes for tetracycline (*tet* (A)), trimethoprim (*dfrA1*, *dfrA5*, *dfrA14*, and *dfrA17*), β-lactam antibiotics (*bla*OXA-1), ammonium compounds (*catA1*) and aminoglycosides (*strA*, *strB*) by animal PG1 isolates was considerably lower than that in PG3 (Figure 4, S3), with the rates being 2.62%, 29.76%, 33.75%, 35.63%, 32.39%, 0%, 5.56%, 0% and 0% in PG1 and 50.93%, 99.32%, 74.52%, 71.17%, 70.70%, 100%, 100%, 49.95% and 49.95% in PG3. The carriage rate of AMR genes by animal PG3 isolates was similar to that in the Chinese human PG3 isolates (Figure 4, S3).

### Host-associated accessory genes

We assessed whether there were any accessory genes were associated with animal associated *S. flexneri*, which may contribute to adaptation using Scoary [50]. A 90% sensitivity and 90% specificity were used as the cut off and association analysis was performed for PG1 and PG3, separately. We identified 33 and 12 accessory genes that were significantly associated with animal isolates in PG3 and PG1 respectively (Table S2). However, BLASTn searches indicated that most of these genes were present in human isolates. One gene, ospE2 was confirmed to be present at very different frequencies of 82.35% and 13.36% in animal and human PG3 isolates, respectively. Multiple insertion sequences (ISs) were found to be genuinely associated with animal isolates in PG1 and PG3. A unique contig detected in 11 animal *S. flexneri* genomes (F9, F39, F38, F34, F28, F27, F26, F24, F21, F20, F14) in PG1 was found to be 100% identical to that in *E. coli* plasmid pLF82 [52].

## Discussion

*Shigella* spp., emerged from within *E. coli* multiple times [3] is a human host-specific pathogen, which pose a significant health threat and with no known animal reservoir until recently [5,7]. In this study, we confirmed that animals were colonized by *S. flexneri* as a reservoir. A comparative phylogenetic analysis of the *S. flexneri* genome isolated from humans and animals showed a clear picture of the population structure of genomic diversity, as well as the relationships between geography, temporal signals, and hosts.

After PG3 was introduced into China, the dominant population of *S. flexneri* changed, and PG3 is nearly the only clade currently circulating in the human population in China. The animal *S. flexneri* population contained a mix of the PG1 and PG3 clades in different proportions to those in the current human *S. flexneri* population in China, suggesting that it is most likely that human *S. flexneri* was transmitted to the animal population at different times and then maintained in the animal population. Molecular dating suggested that the PG1 animal lineages date back to the 1940s to 1960s. Thus, it is likely that PG1 entered the animal population when they were predominantly circulating back and then remained in the animal population. The fact that two animal lineages shared a close relationship with human isolates from China from 1950s to 1960s supports this hypothesis. However, one animal lineage shared a close relationship with recent human PG1 isolates. Therefore, it is also possible that the transmission can be recent in either direction, but cannot be differentiated without more data. The PG3 animal isolates were similar to the isolates circulating in humans in recent years, and all of them belonged to ST91 which carried multidrug resistance and some belonged to the recently emerged novel Xv serotype [17], providing more evidence that *S. flexneri* from humans was transmitted to the animal population. However, we could not identify any direct regional transmission events as none of the animal isolates was close to human isolates from the same region. Interestingly some animal lineages contained animal isolates from different provinces. One animal isolate (F15) from Gansu and three animal isolates (F11, F12 and F17) from Shanxi in PG3 grouped together and were closer to each other than to human isolates from Gansu. Overall, our results showed that animals are a reservoir of diverse *S. flexneri* isolates and that animal domestication is a key factor in the spread and host adaptation of pathogens. It seems that there was no animal to human transmission event in the studied population and/or time period. However, it is possible that animals are an important reservoir and many transmit *S. flexneri* to the human population as the number of analyzed samples were small.

The *S. flexneri* isolates from yaks were obtained from Qinghai and Gansu, which are important yak farming provinces in China, where the earliest domestications of yaks occurred [53]. PG1 *S. flexneri* may have entered the yak population a long time ago, likely when PG1 was predominantly circulating in China. Yaks have a restricted distribution in China, mostly in the Qinghai-Tibet Plateau (QTP) and in the adjacent alpine and subalpine areas where human population density is relatively low and yaks have little contact with humans [53,54]. The yak *S. flexneri* may have been well separated from human population for direct transmission in recent years since the *S. flexneri* isolated from humans from these provinces was similar to human isolates from other parts of China than to yak *S. flexneri*. It would be interesting to conduct sampling of yaks in the less populated regions to further understand the *S. flexneri* evolution in the yaks. Further studies are also needed to determine any transmissions between yak and human populations.

We attempted to determine the genes involved in *S. flexneri* adaptation to the animal population. By analyzing PG1 and PG3 isolates separately, we found that 12 genes in PG1 and 33 genes on PG3 were significantly associated with animal isolates. However, further analysis of the genes revealed that most of these genes were present in human isolates and some were at lower proportions. The initial difference detected was likely because that these genes were not annotated as functional genes. These genes may have continued their degradation as *Shigella* genomes are known to contain many pseudogenes. IS expansion and the associated genome decay is a typical feature of host adapted pathogens [55]. Whether any of the functional genes found to be differentially present in animal isolates are necessary for *S. flexneri* to live in animals remains to be determined. Very few genes that were common to PG1 and PG3 animal isolates were found, suggesting that even if these genes play an adaptive role, they are not essential for surviving in animals. Interestingly, 11 yak isolates belonging to the same lineage in PG1 carried an *E. coli* plasmid LF82, suggesting that this plasmid may have been acquired by their MRCA.

The ability of *Shigella* to cause bacillary dysentery has been attributed to its virulence factors, which are encoded on chromosomal pathogenicity islands and the 210 kb plasmid pINV [13]. Virulence plasmids can provide *S. flexneri* with a competitive advantage to adapt to specific econiches and enhance their survival [56]. The *Shigella* T3SS delivers effectors into the host cells and mediates entry of the bacterium. However, pINV or the invasion region on the plasmid is known to be unstable [57]. During in vitro growth, an increased growth rate of *S. flexneri* happens after lose the PAIs or the entire plasmid, which highlighting the fitness cost of pINV to *S. flexneri* [58]. The presence of the invasion region or the entire plasmid markedly differed between different PGs, suggesting the differences in the stability of the plasmid or invasion region in different PGs. Over 80% of PG1 isolates lost the plasmid or the invasion region but only 18% of the PG3 isolates lost these. The animal PG1 strain has lost the key virulence determinants similar to human PG1 isolates, meaning there is less of a threat of it spreading back to the human population. PG1 is an older lineage than PG3. It is thus possible that the inherent instability of the plasmid may have led to the disappearance of PG1. On the other hand, the increased stability and acquisition of other virulence factors such as SHI-1 may have enabled PG3 to expand globally. Nearly all recent isolates in China belonged to PG3 of ST91 which carried two drug resistance islands and an O antigen modifying plasmid [17]. Acquisition of new drug resistance and the the more stable pINV plasmid may have given PG3 a competitive advantage to replace other PGs in China and can facilitate successful clonal expansion in most Asian and African countries [59].

Animals were not considered a natural host of *Shigella* and the isolation rate from animals was low at 1.62%. Over 60% of the isolates were obtained from calves with diarrhoea, suggesting that *S. flexneri* can cause disease in young animals. Further studies are required to determine the virulence factors needed for them to cause disease in animals. Nearly all PG1 animal isolates lacked the invasion region or the entire pINV plasmid, suggesting that other virulence factors may have played a role in causing diarrhoea.

Examination and comparison of clades within the phylogeny of this lineage revealed different evolutionary rates between humans and animals. Our results suggested animal-associated strains evolve at a considerably slower rate than human-associated strains. It could be caused by multiple factors, such as different selective pressure suffered between the animal and human isolates and insufficient temporal signal in the animal isolates. It is thus plausible that these lineages, once entered the animal population, had evolved slower in the new host environment. The differences of animals in life-history traits may leave a measurable imprint on the isolates’ rate of evolution. However, the temporal signals in animal only isolates from both PG1 and PG3 datasets were insufficient for molecular dating (R^2^ was negative [data not shown]). The lack of temporal signals was likely to have been caused by the limited number of animal isolates sampled and a very short sampling time of only two years (2016–2017) with no animal isolates from any earlier years. Another possibility is that the tip date calibration underestimated the divergence of animal lineages. In particular, PG1 arose earlier than PG3 and had evolved for a longer evolutionary time with deeper branches. Underestimation of deep divergence times based on tip date calibration has been discussed recently in measles virus evolution [42,60]. Long term substitution rates are affected by long-term purifying selection and possible substitution saturation [42]. Therefore, although our results strongly support a rate slow-down in animal isolates, further investigation using a dataset with animal strains sampled over a longer time period are needed to understand any possible effects of transmission between the two different hosts on the evolutionary rates.

In conclusion, there were multiple *S. flexneri* lineages in the animal populations and animals may act as a reservoir of *S. flexneri*. Different clades of *S. flexneri* may persist in the animal population. In particular, *S. flexneri* may have been circulating in the yak population for a long time or transmitted to the yaks during PG1 circulation in the human population in China. Other animal *S. flexneri* isolates were likely recent transmissions from human populations indicating that and animals are a potential reservoir for human infections. Further studies are needed to determine the potential transmission of *S. flexneri* from animals to humans as a public health threat.

## Supplementary Material

Supplemental Material

## Data Availability

Sequencing data were submitted to the Sequence Read Archive (SRA) with the project number PRJNA820478 (The strains used are listed in Supplementary file 1).
