# Supplementary material for: Evolutionary and genomic insights into the long-term colonization of Shigella flexneri in animals
Source: Emerg Microbes Infect. 2022 Aug 31;11(1):2069–79. doi: 10.1080/22221751.2022.2109514 (PMC9448383; doi:10.1080/22221751.2022.2109514)
Supplement: Supplemental Material [file TEMI_A_2109514_SM9558.zip › Fig S2.pdf]

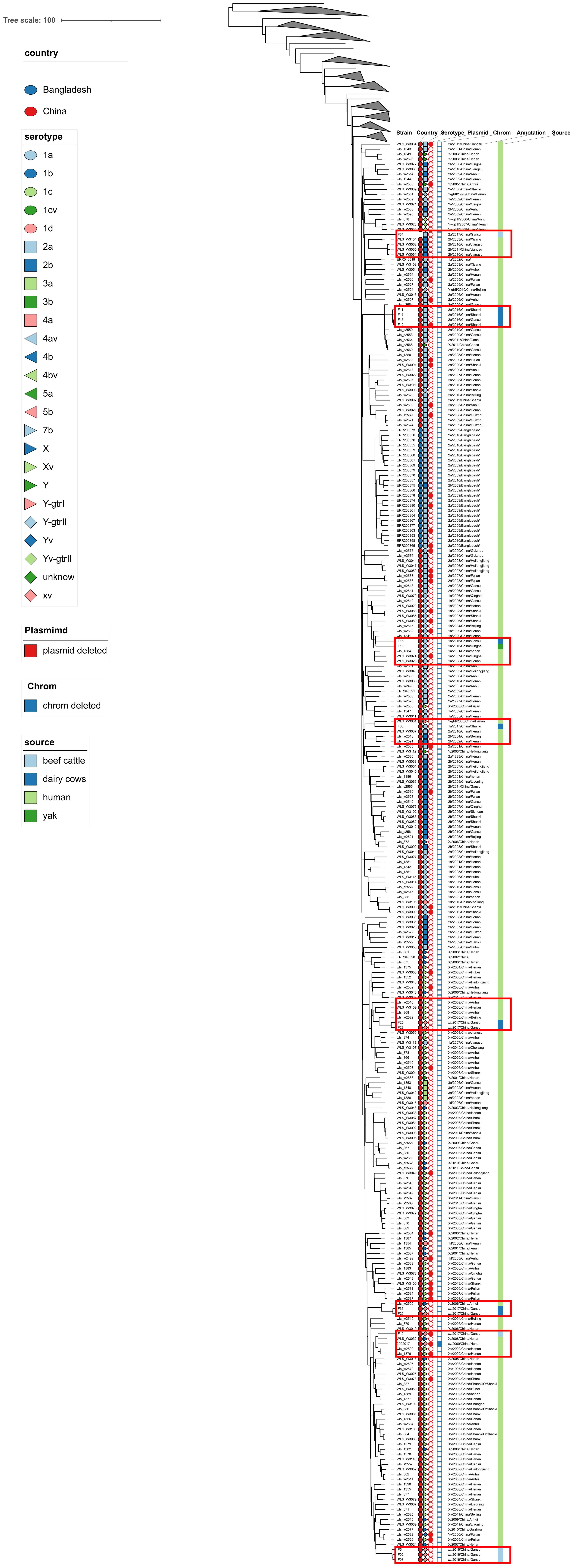
Tree scale: 100
country
Bangladesh
China
serotype
1a
1b
1c
1cv
1d
2a
2b
3a
3b
4a
4av
4b
4bv
5a
5b
7b
X
Xv
Y
Y-gtrI
Y-gtrII
Yv
Yv-gtrII
unknow
xv
Plasmimd
plasmid deleted
Chrom
chrom deleted
source
beef cattle
dairy cows
human
yak
Strain
Country
Serotype
Plasmid
Chrom
Annotation
Source
