# Supplementary figures and images for: Evolutionary and genomic insights into the long-term colonization of Shigella flexneri in animals
Source: Emerg Microbes Infect. 2022 Aug 31;11(1):2069–79. doi: 10.1080/22221751.2022.2109514 (PMC9448383; doi:10.1080/22221751.2022.2109514)

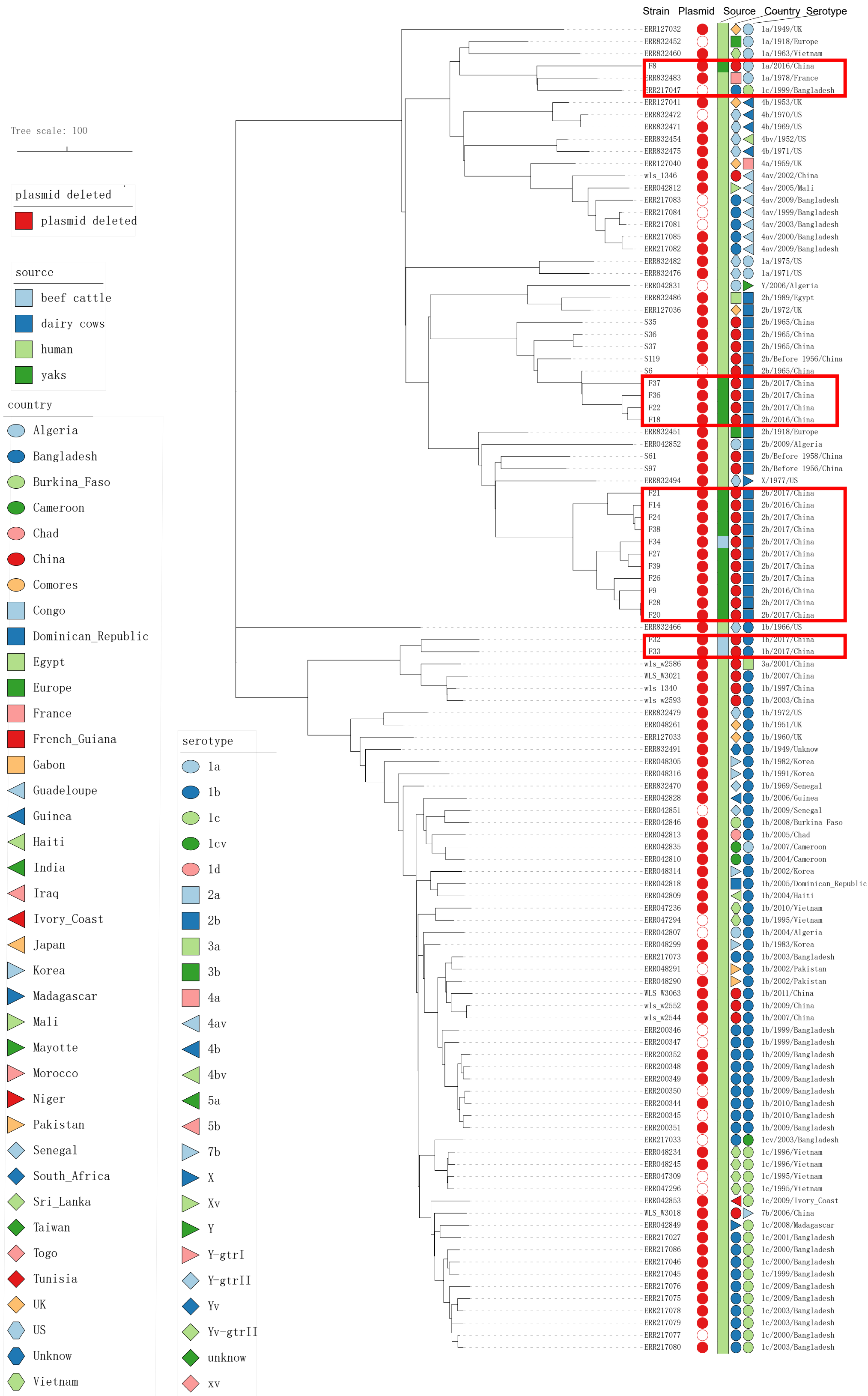

Supplement: Supplemental Material [file TEMI_A_2109514_SM9558.zip › Fig S1.pdf]

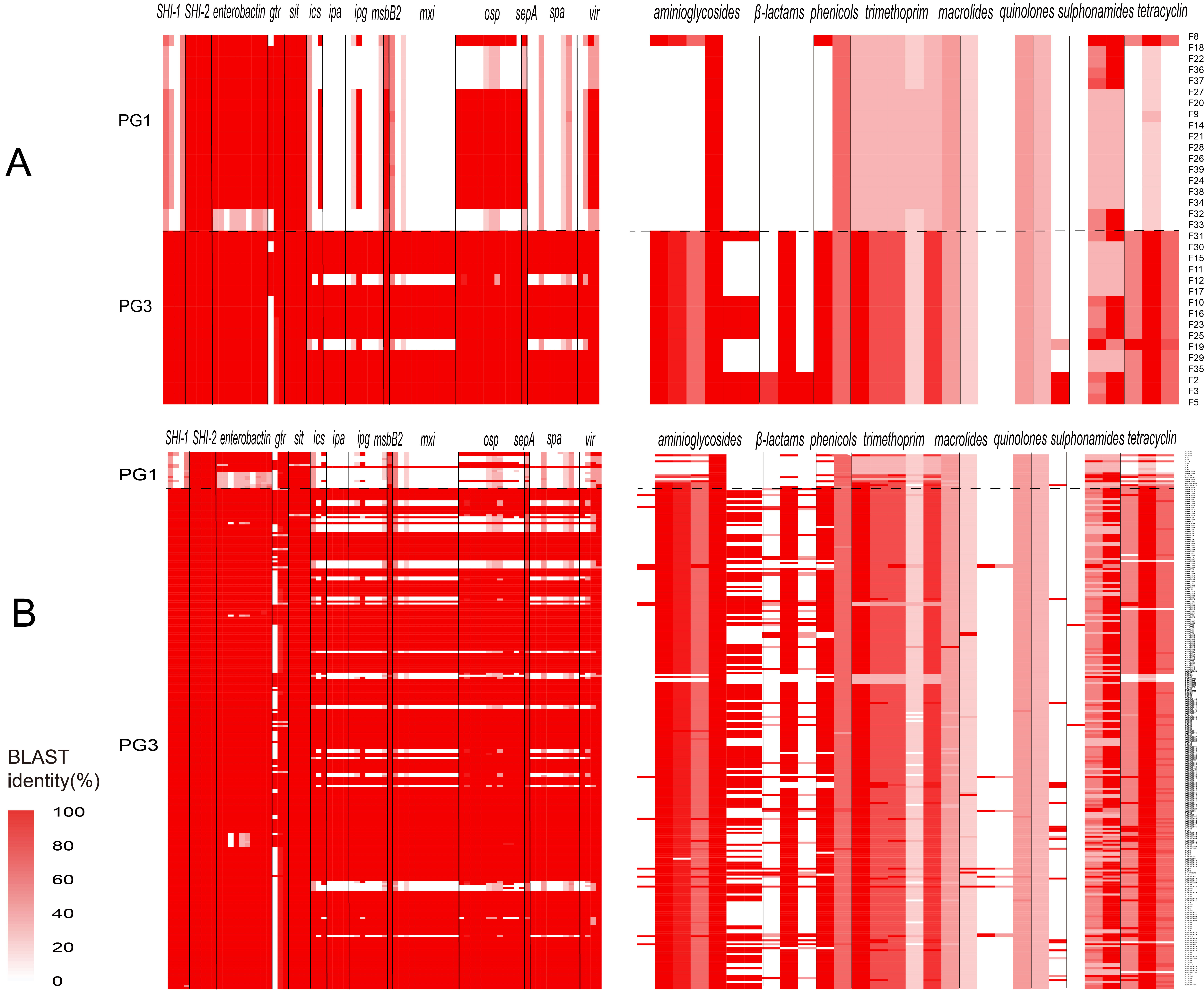

Supplement: Supplemental Material [file TEMI_A_2109514_SM9558.zip › Fig S3.jpg]
